# Do Histories of Painful Life Experiences Affect the Expression of Empathy Among Young Adults? An Electroencephalography Study

**DOI:** 10.3389/fpsyg.2021.689304

**Published:** 2021-07-16

**Authors:** Parvaneh Yaghoubi Jami, Hyemin Han, Stephen J. Thoma, Behzad Mansouri, Rick Houser

**Affiliations:** ^1^Educational Psychology Program, University of Alabama, Tuscaloosa, AL, United States; ^2^Department of Curriculum and Instruction, University of Alabama, Tuscaloosa, AL, United States; ^3^Counselor Education Program, University of Alabama, Tuscaloosa, AL, United States

**Keywords:** empathy, ERP, physical pain, psychological pain, similarity

## Abstract

Previous research suggests that prior experience of pain affects the expression of empathy. However, most of these studies attended to physical pain despite evidence indicating that other forms of pain may also affect brain activity and emotional states in similar ways. To address this limitation, we compared empathic responses of 33 participants, some of whom had experienced a personal loss, across three conditions: observing strangers in physical pain, psychological pain, and a non-painful condition. We also examined the effect of presence of prior painful experience on empathic reactions. In addition, we examined the stimulation type, prior experience, and ERPs in the early Late Positive Potential (300–550 ms), late Late Positive Potential (550–800 ms), and very late Late Positive Potential (VLLPP; 800–1,050 ms) time windows. Behavioral data indicated that participants who had personally experienced a loss scored significantly higher on perspective taking in the psychological-pain condition. ERP results also indicated significantly lower intensity in Fp2, an electrode in the prefrontal region, within VLLPP time window for participants experiencing a loss in the psychological-pain condition. The results of both behavioral and ERP analysis indicated that prior experience of psychological pain is related to cognitive empathy, but not affective empathy. The implication of these findings for research on empathy, for the study of psychological pain, and the moderating influence of prior painful experiences are discussed.

## Introduction

Historically, empathy is considered an automatic emotional response that leads to either “self-oriented”—known as personal distress—or “other-oriented”—known as empathic concern—feelings (Cuff et al., 2016). These two expressions of empathy are also differentially related to altruistic behavior (Endresen and Olweus, 2001). Nevertheless, such one-dimensional ideology has changed toward a more complex, multidimensional perspective (Decety and Hodges, 2004; Fan and Han, 2008) supporting the integration of affective and cognitive processes in empathic behavior. For example, Fan and Han (2008) observed an activity over the frontal-central lobe around 140 ms stimulus onset followed by a later activity (at 380 ms after stimulus presentation) over the central-parietal region, while participants were witnessing others’ pain. Similarly, fMRI studies identified the activation of distinct brain areas in limbic system [anterior insula (AI) and dorsal anterior cingulate cortex (dACC)] and the medial prefrontal cortex in empathic-inducing situations. According to these results, the aforementioned regions showed stronger activity in empathic-inducing conditions compared with control conditions (Rameson et al., 2012; Morelli et al., 2014) suggesting that empathy is an integration of bottom-up automatic and top-down cognitive responses. Following the results of these studies (see Cuff et al., 2016, for review), the current paper defines empathy as a multidimensional concept consisting of three distinct, but interrelated, components, namely, empathic concern, personal distress, and perspective taking (Davis, 1983).

The empirical literature informing our understanding of empathic behavior is derived from studies that compare people’s reaction after receiving pain versus observing others’ pain. More specifically, these studies assess observers’ feelings while receiving pain or witnessing others in pain using different methodologies, especially neuroimaging methods, to better understand the mechanism of empathy (Eisenberger et al., 2003; Singer et al., 2004; Lamm et al., 2010). Although, in most of these studies, physical pain was used to trigger empathic responses, a more comprehensive review of the pain literature indicates that there are three types of pain: physical pain resulting from tissue damage, such as acute injury, social pain associated with losing social bonds as the result of social isolation/rejection, and psychological pain associated with the loss of a loved one through death, divorce, or a relationship break up (Mee et al., 2006; Eisenberger, 2012). Depending on the type of pain assessed, results suggest that the suffering person will sense either the pain and the associated emotions or a lesser experience of an unpleasant feeling without actually sensing the pain (MacDonald and Leary, 2005; Jaremka et al., 2011). Because of the growing interest in the distinctiveness of psychological pain, the focus of this study is on empathic responses toward observing others’ psychological pain.

### Empathy and Physical Pain

Neuroscientific discoveries have broadened the understanding of functional neuroanatomy of physical pain, particularly through the development of a pain matrix consisting of two distinct, but interrelated, components. The affective component of the matrix mostly relates to activity in the dACC, cerebellum, and the anterior insula, while the primary somatosensory cortex (S1), secondary somatosensory cortex (S2), and posterior insula (PI) are associated with the sensory component (Peyron et al., 2000; Wager et al., 2013). In this view, a person senses pain through activation of the sensory component of the pain matrix. Similarly, through activation of affective component, the person feels unpleasant emotions associated with the pain (see Eisenberger, 2012, for discussion).

Numerous studies have shown that observing others’ physical pain activates the affective facet of the pain matrix. That is, the empathizer cannot sense the pain of the suffering person; however, they can feel the negative emotional reactions of the pain (Wicker et al., 2003; Lloyd et al., 2004; Singer et al., 2004; Botvinick et al., 2005; Jackson et al., 2005; Bufalari et al., 2007; Decety et al., 2008).

### Empathy and Social Pain

Unlike physical pain, experiencing social pain activates the affective facets of the pain matrix. In other words, being socially rejected/excluded is associated with the unpleasant emotions of pain rather than sensing the pain (Eisenberger et al., 2003; Eisenberger and Lieberman, 2004; O’Connor et al., 2008; Bolling et al., 2011). On the other hand, observing others’ social pain activates brain areas involved in mentalizing (i.e., dorsal medial prefrontal cortex, precuneus, and temporal pole; Meyer et al., 2012).

### Empathy and Psychological Pain

Current evidence suggests that psychological and physical pains are experienced similarly (Woo et al., 2014). Thus, suffering from psychological pain would activate the whole pain matrix (Kross et al., 2011). Previous studies also explored empathic response toward others’ psychological pain (i.e., grief) and reported activation in middle and posterior cingulate gyrus, the inferior frontal gyrus, the middle temporal gyrus, the thalamus, and the brainstem (Gündel et al., 2003; O’Connor et al., 2008; Kersting et al., 2009).

### Summary

To summarize, people experiencing physical or psychological pain not only *sense* the pain, but also *feel* the unpleasant emotions associated with it, whereas being in social pain is associated with only having negative feelings of the pain (Eisenberger et al., 2003; Singer et al., 2004; O’Connor et al., 2008; Kross et al., 2011). Similarly, observing others suffering from physical or psychological pain also differs from observing others in social pain. In the case of the first two types of pain and depending on the context, either the whole pain matrix (psychological pain) or only affective facet of pain matrix (physical pain) would be activated for an empathic reaction (Damasio et al., 2000; Meerwijk et al., 2013). On the other hand, responding empathically after observing another individual being rejected/excluded from their social group requires cognitive processes (mentalize the suffering person’s situation) rather than the activation of the pain matrix (see Yaghoubi Jami et al., 2021, for review). It should be emphasized that these three types of pain are inter-connected in everyday life, given that psychological pain is likely interwoven with social relations of various kinds.

In addition, it should be noted that regardless of the type of pain people are witnessing, empathic behavior is moderated by the relationship between the empathizer and the target of empathy (Twenge et al., 2007; Xu et al., 2009; Mathur et al., 2010; Beeney et al., 2011), or having a shared painful experience with the target of empathy (Eklund et al., 2009). The following section is a review of studies exploring the links between empathic behavior and empathizer’s personal experience of the observed pain.

### Prior Painful Experience and Empathic Response

Most of us have heard the phrase, “*I’ve been there too”* referring to undesirable experiences, such as grief. The literature suggests that sharing similar painful experiences is a predictor of empathic responsiveness (Batson et al., 1996; Eklund et al., 2009; Preis and Kroener-Herwig, 2012). For example, Barnett et al. (1987) investigated the association between empathy and similarity by asking participants to listen to a conversation between a rape victim and her therapist. According to the result, those participants with an experience of abusive behavior showed higher empathy toward the victim. The reported results were confirmed by other studies (Barnett, 1984; Barnett et al., 1986).

Hoffman (2001) clarified the rationale of the relevance of similarity for empathy by highlighting the important role of memory. Accordingly, encountering another individual in a situation similar to the one experienced by the empathizer evokes the memory of the prior experience and elicits emotions similar to the observed individual. For example, if an individual has experienced the loss of a loved one and later observes another individual in mourning, the past experience of grief facilitates the affective and cognitive interpretation of the grieving person’s situation.

According to this brief review, similarity of painful experience – whether it be physical or psychological – is important for empathic responses. The effect is observed primarily when the observed pain refers to *similar painful experiences* rather than drawing attention to similarities between the observer and observee. For this reason, similarity is treated as a similar prior *painful experience* in the present study.

## Purpose of the Study

Although many research traditions assess empathic reactions toward others’ physical and social pain, there is a dearth of research on the mechanisms and perceptions of psychological pain (see Meerwijk et al., 2013, for review). Moreover, most of the studies in the realm of empathy and pain are either behavioral or fMRI studies. What remains unaddressed in the literature is exploring the temporal aspects of empathic responsiveness in psychologically painful situations. The literature suggests that an accurate understanding of psychological pain could lead to preventing its dire consequences, such as mental health breakdown or even suicide (Bosticco and Thompson, 2005; Mee et al., 2006). This knowledge could not be gained without studying how this type of pain is perceived and processed in the brain. Additionally, there is a lack of conclusive evidence on the individual characteristics contributing to empathic behavior.

To circumvent the issues that have challenged previous researchers, this study focused on the association between prior experience of psychological pain in form of grief and associated empathic responses. To achieve these goals, the current paper employed EEG to investigate neural correlates of people’s reaction to observing others’ pain with and without apparent signs of pain (physical and psychological). In addition, the study assesses the feelings and reactions that might be aroused by attending to observed person. Moreover, to clarify the relationship between similarity and empathic reactions, the authors assessed the impact of being familiar with a possible psychological pain-inducing situation on empathic behavior.

Prior studies assessing neural correlates of empathy have reported significant differences in prefrontal regions across painful and non-painful conditions across subject groups (Decety and Jackson, 2004; Light et al., 2009; Leigh et al., 2013; Reniers et al., 2014). Thus, although our study is primarily exploratory, we hypothesized that significant effects of the condition and prior experience of psychological pain would be found in frontal regions electrodes. Whole-brain analysis was also performed for further exploration. In terms of time windows of interest, we focused on relatively late components of ERPs (e.g., 500 ms and even 1,000 ms) given previous EEG/ERP studies suggesting such late components were useful indicators to examine between-person and between-condition differences (e.g., Hajcak et al., 2010; Blanchette and El-Deredy, 2014). In addition, for exploratory purposes, we examined correlations between participants’ ERPs, behavioral responses, and dispositional empathy.

## Materials and Methods

### Participants

A total of 41 undergraduate college students (six males; *M* = 19.50, *SD* = 1.39, all right-handed) participated in this study. Although we could not estimate the required sample size based on power analysis due to the lack of relevant previous studies and because of exploratory nature of our study, we referred to similar social neuroscientific ERP studies (Fan and Han, 2008; Blanchette and El-Deredy, 2014). These previous studies were conducted with 16 to 31 participants; therefore, we recruited 41 participants after considering potential exclusions.

Participants consented to be part of the study and were compensated by receiving a course credit. The study was approved by the Institutional Review Board of a southern university in the United States and was performed in accordance with the Declaration of Helsinki (1964) and its later amendments. The primary inclusion criteria were age (18 to 22 years) due to developmental path of empathy (Yaghoubi Jami et al., 2018), having no self-reported history of any psychiatric diagnoses and no uncorrected vision deficits.

To ensure eligibility, interested participants provided information about their medical conditions and any history of neurological disorders using a short medical history form. The medical form was used to identify participants for whom the procedures may be of risk (e.g., prone to seizure and taking particular medicine). Participants who failed the screening test (*N* = 1) were compensated and dismissed.

After removing incomplete responses or responses in which participants had excessive motion causing artifacts on neural recording, the analyses were performed on 33 participants.

### Stimuli and Apparatus

The experiment had two parts: an online self-reported questionnaire, Interpersonal Reactivity Index (IRI; Davis, 1983), measuring dispositional empathy as a multidimensional concept (affective and cognitive facets), and a computer-based task. Affective empathy and cognitive empathy were measured through *empathic concern* (EC), and *perspective taking* (PT), items, respectively. *Personal distress* (PD) items were used as a separate measurement of personal distress. Because of debates over the validity of the *fantasy scale* (FS) items in measuring empathy (Yaghoubi Jami et al., 2018), this subscale was not included in the analysis. Following Davis’ (1983) guidelines, items were scored on a 5-point Likert scale (0 = *does not describe me well* to 4 = *describe me well*—some items were reverse-coded) with a final score range of 0 to 28 on each scale. Higher score on each scale indicates higher tendency on that subscale (e.g., score of 28 on *empathic concern* scale suggests participants reported themselves as having high affective empathy). The IRI showed acceptable internal consistency reliability: *α*_empathic concern_ = 0.79, *α*_personal distress_ = 0.69, and *α*_perspective taking_ = 0.75.

Additionally, to assess the degree of prior psychological painful experience, five questions were presented to participants asking about their personal experience of loss. Following the self-disclosed loss experience, participants were asked: How close was the person to you? (1: “*Very close*” to 5: “*Not at all*”), How severe was the loss for you? (1: “*Extremely painful*” to 5: “*Not painful at all*”), How did you feel immediately after the loss? (1: “*Desperate/Depressed*” to 5: “*No feeling*”), Do you still think about that person? (1: “*Always*” to 5: “*Never*”), and How do you feel now? (1: “*Desperate/Depressed*” to 5: “*No feeling*”). This researcher-constructed questionnaire showed acceptable reliability (*α* = 0.80). Based on these responses, any participant who did not have a first-hand experience of grief, or who reported that they did not have a relationship with the person, barely thought about them, and did not have any feelings after losing them, were identified as the *No-Loss* group (*N* = 15). By contrast, participants in the *Loss* group (*N* = 18) reported to have lost a (very) close relative, felt (very) much pain after their loss, thought about the deceased person frequently, and were still feeling (very) sad because of their loss. Demographic backgrounds did not differ significantly between the two groups (*p* > 0.05).

#### Visual Stimuli

**Figure 1:**
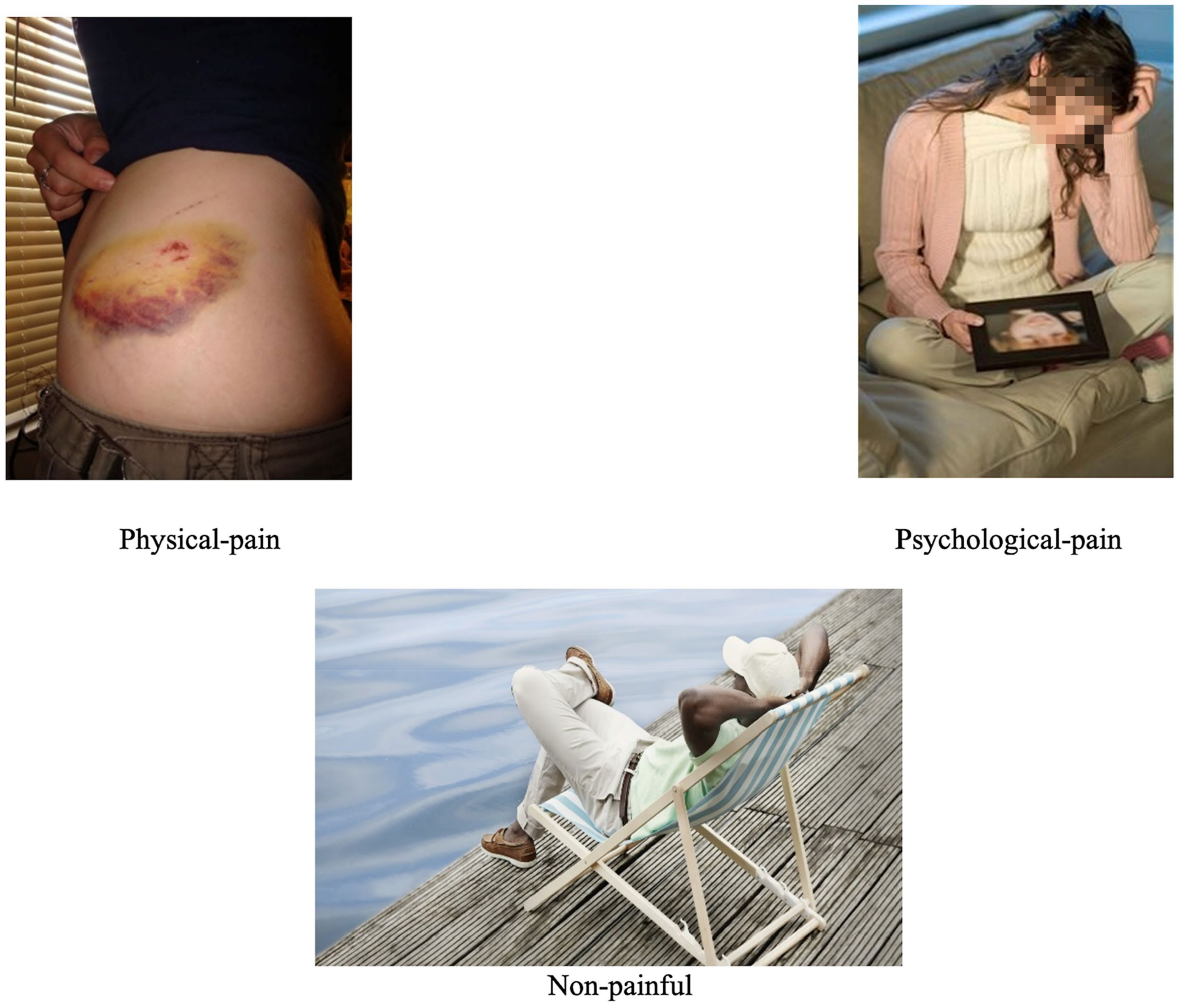
Stimulus examples.

In a previous study, a picture database was created and validated in order to record participants’ empathic reaction to various situations (Yaghoubi Jami et al., 2021). Specifically, the picture database was created using a series of steps. First, a pilot study was conducted using 90 pictures which depicted painful physical (e.g., needle injection) and psychological (e.g., grieving mother) situations as well as non-painful incidents (e.g., happy babies). These pictures were selected using an online search engine (Google image search) and were grouped into three categories (i.e., physical pain, psychological pain, and non-painful) based on the keyword used for the initial search. As part of this measure development stage, volunteers (both male and female; *N* = 90) varying in age, educational level, and nationality rated the pain intensity of the pictures on a 5-point scale ranging from *very painful* (1) to *not at all* (5). Based on participants’ rating, pictures that received an average ranking between *very painful* to *moderately painful* were placed into one of two painful conditions: physical-pain (*N* = 7): *M* = 1.89, *SD* = 0.41, and psychological-pain (*N* = 7): *M* = 1.54, *SD* = 0.46. The selected pictures in each category showed an acceptable internal consistency reliability: *α*_physical-pain_ = 0.68, *α*_psychological-pain_ = 0.79. For the non-painful condition, seven pictures ranked by the same participants as *not at all painful* were selected. The pictures were matched based on gender (three males, three females, and one child), number of people in the frame, face or faceless, and picture size (800 × 600 pixels) between conditions. The final database consisted of 21 color pictures representing strangers in physical pain, psychological pain, and non-painful conditions (Figure 1). To ensure that participants interpreted the pictures in the intended way, all pictures were labeled by the associated category so that each participant always viewed a picture *plus* a category label; for example, a picture of a man standing in a graveyard was labeled as “psychological pain.” The categorization of pictures used in this study was supported by a previous study in which 91% of participants used similar labels for the pictures. The manipulation used in the current study was further supported in two additional studies (Yaghoubi Jami et al., 2021).

The stimuli were presented using Presentation Software.^1^ All pictures were presented in the center of a gray background on a 15-inch CRT monitor. Participants viewed the display from a distance of 60 cm and used an external numeric keypad (that was placed next to them) to press the appropriate button (ranging from 1 to 5) for responding to questions following each picture (see Section “Procedure”).

#### EEG Recording

Continuous EEG was recorded from 21 active electrodes using MITSAR EEG system with an amplifier model 201 (Mitsar Co., Ltd., St. Petersburg, Russia; distributed by Nova Tech, Inc., Mesa, AZ, United States). For recording EEG signals, a 19-channel ElectroCap electrode cap (Electro-Cap International Inc., Eaton, OH, United States) was placed on participant’s scalp based on the international 10–20 system (American Electroencephalographic Society, 1994). The other two electrodes (A1 and A2) were placed in left and right ears. The ground electrode was placed on the forehead. Following the system manual and previous studies using the same device (Kropotov et al., 2005), impedance was kept below 10 kΩ. EEG signals were collected at a sampling rate of 512 Hz and processed online with the Mitsar EEG Acquisition software using a Dell XPS 15 laptop. EEG data were referenced online at vertex electrode (Cz) and re-referenced again using mastoids. To remove any possible contamination of muscle artifacts, appropriate filters were applied (100 Hz low-pass, 0.5 Hz high-pass, and 60 Hz notch filters) and every trial was inspected visually.

### Procedure

Three days after completing the IRI, participants were scheduled for the recording session. Upon participants’ arrival to the laboratory, they were informed about the procedure and signed the written consent. To avoid social desirability bias (Eklund et al., 2009), participants were debriefed about the real aim of experiment at the end of the study.

The study started with participants filling out the medical history form. Eligible participants (i.e., those who passed the screening) received demographic and researcher-made questionnaires. While participants were completing the two mentioned questionnaires, experimenters prepared participant for the study (putting the EEG cap, connecting the electrodes, connecting the amplifier to the electrodes and stationary computer, checking the signals, and so on). The experiment was held in a quiet room with a sufficient and quiet air conditioner. Before data recording, one of the researchers explained the procedure and underwent a training session with the participant to become familiar with the study. Specifically, participants observed a picture from one of the conditions (selected randomly) and answered subsequent questions, while the experimenter explained instructions and questions. The practice trial was not recorded. When the participant was comfortable with the procedure and instruction, the experimenter left the room, and the recording session began.

**Figure 2:**
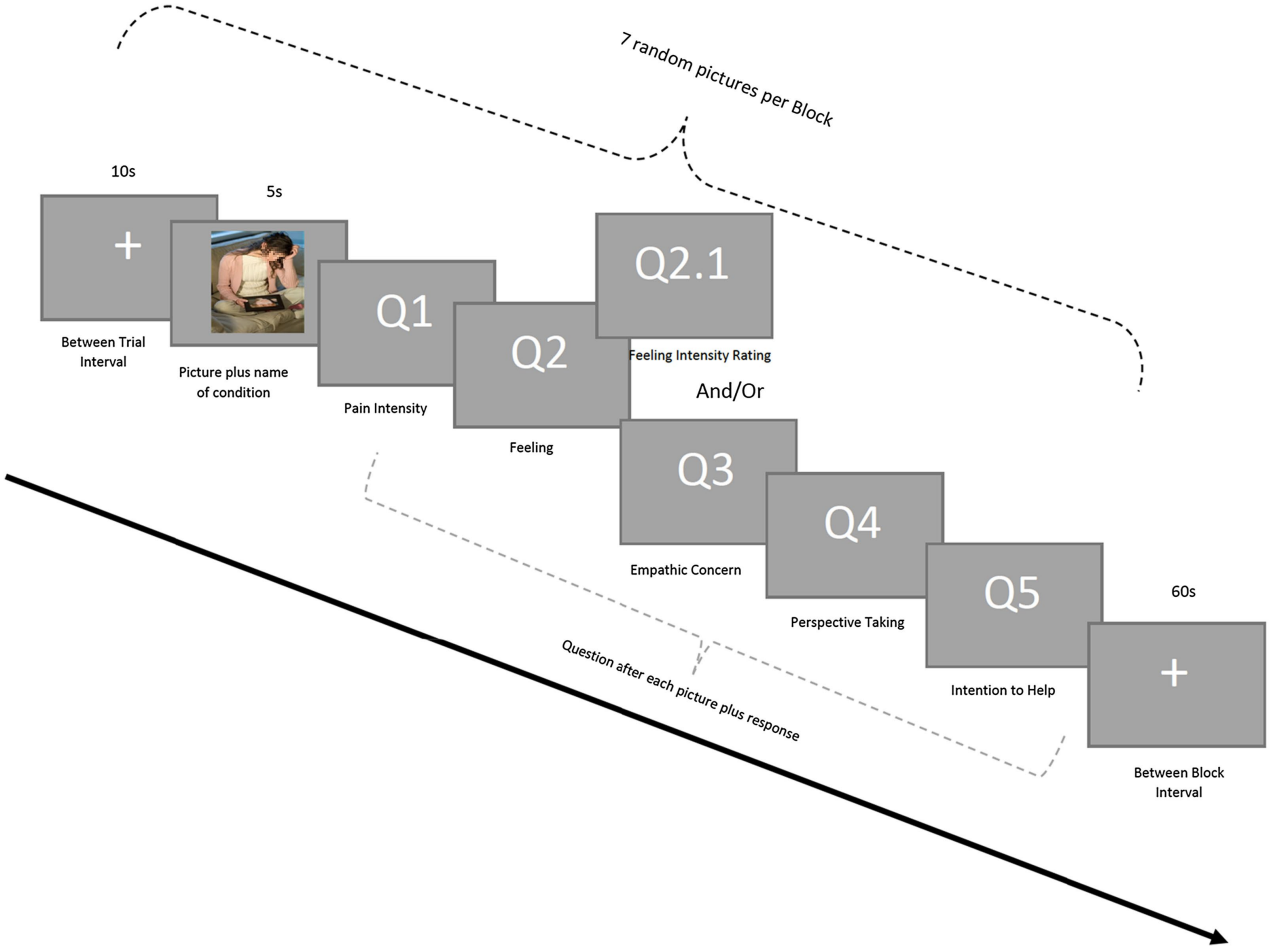
Experimental procedure.

Participants were presented with pictures showing a person in one of three conditions: non-painful, physical-pain, and psychological-pain. In total, the experiment consists of three blocks of seven pictures with 60-s inter-block intervals. To reduce exhaustion and allow participants to move freely without disturbing the data, a fixation mark was displayed on the screen for 10 s between the trials. Participants observed each picture for 5 s and used an external keypad to answer questions regarding their **pain intensity** (1: *very pain* to 5: *no pain*), **feeling** (1: *sad*, 2: *distress*, 3: *no feeling*, and 4: *happy*), **empathic concern** (1: *extreme empathic concern* to 5: *no empathic concern*), **perspective taking** (1: *can imagine* to 5: *no understanding of the situation*), and **intention to help** (1: *definitely help* to 5: *will not help*) on a 5-point Likert scale. To avoid ordering effect, the questions were counterbalanced across the trials. See Figure 2 for the visualized description of the procedure.

The pictures were shown randomly to balance participants’ negative emotions that could have been elicited by pictures. In case of any emotional breakdown or feeling uncomfortable resulted from observing pictures, participants could have a session with a counselor who was sitting in the other room. To ensure task compliance, participants were told that each session was being recorded and they might be selected for follow-up questions about the pictures. The videotapes were deleted after each session. The whole data recording lasted on average 21.55 (*SD* = 3.69) minutes.

### Data Analyses

#### Behavioral Data Analysis

To compare dispositional empathy between the two groups, an independent t-test was used. Next, to assess the effect of condition and participants’ group affiliation and any interaction between the two on participants’ reactions, we conducted a doubly multivariate analysis of variance (MANOVA) with condition as the within- and group as the between-subject factors. Doubly MANOVA was chosen because one of the dependent variables (i.e., participants’ feelings) had a different scaling than the other four dependent variables (Ho, 2006). In case of any significant multivariate effect, the result of univariate ANOVA with Bonferroni correction (*α* = 0.025) and Greenhouse-Geisser correction (whenever applicable) is reported. No univariate or multivariate outliers (*p* < 0.001) were found, and all assumptions (i.e., sampling distributions normality, homogeneity of variance-covariance matrices, and linearity) of doubly MANOVA were met. All analyses were conducted using IBM SPSS, version 24.

#### ERP Analysis

EEGlab software^2^ (Delorme and Makeig, 2004) was used for preprocessing the collected data and analyzing the EEG signal. The analyses were based on computer off-line on stimulus-locked ERPs meaning only trials in which participants observing a picture from one of the conditions were included in ERP analysis. All trials in which participants were required to provide a response (e.g., rating pain intensity) were excluded from ERP analysis and included in behavioral analysis.

The collected data were first filtered with a high-pass filter (lower edge frequency) at 1 Hz. The filtered dataset was re-referenced by using Cz as the reference channel following the EEGLAB tutorial manual (Delorme et al., 2007). The EEG data were then epoched from 100 ms before the stimulus (for the baseline correction) and 1,500 ms after the stimulus. The epoched dataset was further processed with the independent component analysis (ICA) for artifact removals. For the ICA, the *runica* algorithm implemented in EEGLAB was used. In short, EEG recording data are a mixture of the source signal. By applying ICA filters, we can produce the maximally temporally independent signals available in the channel data. ICA components are temporally distinct even when their scalp maps are overlapping. With the help of ICA, we can determine whether a signal is an artifact (e.g., muscle or eye movement) or it is cognitively related. After applying ICA, components are ordered based on their contribution to the original signal. Therefore, movement artifacts can be detected as they have the strongest contribution (Delorme and Makeig, 2004; Delorme et al., 2007).

Following the relevant literature (Kropotov et al., 2005; Cheng et al., 2014), using WinEEG Advanced Software,^3^ any trial having electrooculogram artifacts exceeding ±100 μV threshold were excluded from analysis. Additionally, trials containing eye or muscle movements were excluded from the analysis.

Once all artifacts were removed from the dataset, ERP were compared across groups and conditions within three time-windows: the early Late Positive Potential (ELPP), late Late Positive Potential (LLPP), and very late Late Positive Potential (VLLPP) time windows. The ELPP was defined 300–550 ms after each response. The LLPP was defined 550–800 ms after each response. The VLLPP was defined 800–1,050 ms after each response. We focused on these three time-windows as they are reported to be significantly involved with development of emotional regulation and reappraisal (Hajcak et al., 2010; Blanchette and El-Deredy, 2014; Cheng et al., 2014).

We calculated the mean ERP within the aforementioned time windows with a customized MATLAB script. It should be noted that several previous studies have utilized other EEG/ERP indicators, such as the peak latency and the amplitude of each individual ERP, in their analyses. However, we decided to use the time window-specific mean because it has found to produce less biased analysis outcomes; in fact, the supporters of use of the mean ERP have argued that the use of peak components should be avoided due to its relatively greater bias and worse efficiency (Luck, 2005; Clayson et al., 2013).

For statistical analysis, we first performed a mixed-effects analysis for each time window. This statistical analysis was conducted using a customized R script. Because responses were nested within each participant, we set the mean ERP as the dependent variable, the electrode location, condition, and group as the fixed effects, and participants’ ID and trial numbers as the random effects. We also entered the two- and three-way interaction effects among the electrode location, group, and condition, into the model as fixed effects. The mixed-effects analysis was performed with an R package, *lme4*. The aforementioned analyses, including both the mixed-effect analyses and electrode-wide comparisons, were performed within the hypothesized regions, frontal regions, and the whole brain. For the region-specific analyses, Fp1, Fp2, Fz, F7, F3, F4, and F4 were included. We intended to focus on the frontal regions since previous studies have shown that activity in the regions was significantly associated with empathic responses and pain perception (Rameson et al., 2012; Morelli et al., 2014). These seven electrodes are associated with the frontal regions (Minnerly et al., 2019) and were used as the foci of statistical analyses.

Moreover, we compared ERP for each electrode to examine the effects of group affiliation and condition as well. We performed additional mixed-effects analysis while setting ERP in each electrode as the dependent variable. Similar to the whole-brain analysis, in this analysis, participants’ ID and trial numbers were used as the random effects, and the group and condition were used as the fixed effects. We also examined the group and condition interaction effect. In this process, in order to deal with the issue associated with false discovery rate during multiple tests, we adjusted the false discovery rate to *q* = 0.05 by using an R package, *fdrtool*. Once we found significant effect(s) within a specific electrode, we plotted ERP per group and condition for visual demonstration. In addition, we performed additional *t*-tests to examine differences between groups across conditions as well.

#### Exploratory Correlational Analysis

To explore the relationships between participants’ behavioral responses, dispositional empathy, and ERPs, we conducted correlational analyses. Prior to this analysis, we first calculated the mean of each of the behavioral responses recorded as continuous variables, the pain perception, empathic concern, perspective taking, and intention to help, per condition per participation. Second, we calculated the mean of the ERP in each electrode per condition per participant. Third, dispositional empathy scores in terms of IRI-EC, IRI-PT, and IRI-PD per participant were used.

The large number of variables associated with the correlational analysis raised the possibility of false positives. To address this concern, we performed a Bayesian correlational analysis. According to Bayesian methodologists, the focus of a statistical analysis does not depend on values of *p* and the rejection of null hypotheses. By contrast, Bayesian analyses attend to the extent to which the obtained evidence supports the presence of a significant effect (Han et al., 2018; Wagenmakers et al., 2018). This shift in focus buffers Bayesian analysis from the possibility of false positives due to multiple test (Gelman et al., 2012).

Bayesian correlational analysis was performed with *BayesFactor* package in R (Morey et al., 2018). The analysis was performed for the *physical-pain* and *psychological-pain* conditions. In the case of the correlational analysis with ERPs, we focused on the time window(s) that showed significant ANOVA and electrode-wise analysis results. While interpreting results, we used the resultant Bayes factor (BF) that indicates to which extent an alternative hypothesis was supported by evidence. We used 2log*BF* ≥ 2 as the threshold indicating the presence of positive evidence (Han et al., 2018; Wagenmakers et al., 2018).

## Results

### Behavioral Analysis

The results of behavioral data analysis are reported based on the order of analytical approach explained in Section “Data Analyses.”

#### Prior Psychological Painful Experience and Dispositional Empathy

There were no significant group differences in the self-reported questionnaire subscales: IRI-EC, *t*(31) = −0.02, *p* = 0.98, *d* = 0.01; IRI-PD, *t*(31) = −1.57, *p* = 0.13, *d* = 0.54; and IRI-PT, *t*(26.57) = −0.09, *p* = 0.93, *d* = 0.03. Regardless of group affiliation, participants’ affective empathy was significantly higher than cognitive empathy, *t*(32) = 4.42, *p* < 0.001, *d* = 0.77. Regarding personal distress, the *Loss* group reported to have less distress compared to their peers in *No-Loss* group; however, the difference was not statistically significant (Table 1).

**Table 1:** Group comparison of dispositional empathy.

	Loss		No-Loss		Value of *p*
	Mean	*SD*	Mean	*SD*	
IRI-EC	20.50	3.59	20.53	4.42	0.98
IRI-PT	17.28	4.87	17.40	2.56	0.93
IRI-PD	10.44	3.01	12.40	4.15	0.13

*Means and standard deviations are reported. IRI-EC, empathic concern; IRI-PT, perspective taking; and IRI-PD, personal distress. There is no difference between the two groups (i.e., Loss and No-Loss) with respect to dispositional empathy*.

#### Prior Psychological Painful Experience and Situational Empathy

##### Condition Comparison

Results indicated a significant multivariate effect of condition on participants’ responses, Wilks’ *λ* = 0.008, *F* (10, 22) = 268.03, *p* < 0.001, *η*^2^ = 0.99. The detailed results for each item are explained below.

##### Pain Intensity

A significant effect of conditions on participants’ pain intensity was found, *F* (2, 62) = 1599.44, *p* < 0.001, *η*^2^ = 0.98. Psychological-pain and non-painful conditions were rated as the most and least painful conditions. Pairwise comparisons indicated significant difference between all conditions (*p*s < 0.025).

##### Intensity of Feelings

Condition had a significant effect on participants’ feelings, *F* (1.654, 51.263) = 24.34, *p* < 0.001, *η*^2^ = 0.44. The distribution of emotion that was aroused by each condition is as follows: physical-pain, distressed (*N* = 17), sadness (*N* = 13), and no feeling (*N* = 3); psychological-pain, sadness (*N* = 31), distressed (*N* = 1), and no feeling (*N* = 1); and non-painful, happiness (*N* = 28), and no feeling (*N* = 5).

##### Empathic Concern

There was a statistically significant difference between conditions with respect to participants’ empathic concern, *F* (1.107, 34.312) = 103.517, *p* < 0.001, *η*^2^ = 0.77. Observing another individual’s psychological pain evoked higher empathic concern than physical pain and non-painful. Expectedly, all conditions differed significantly in the level of empathic concern they aroused (*p*s < 0.001).

##### Perspective Taking

Follow-up analysis results indicated a significant effect of condition on participants’ perspective taking, *F* (2, 62) = 22.67, *p* < 0.001, *η*^2^ = 0.42. As the pairwise comparison indicated, participants reported to be able to imagine or put themselves in the protagonists’ position while observing a stranger in non-painful or psychological-pain situation, which was significantly different from physical-pain condition (*p*s < 0.001). No significant difference was observed between non-painful and psychological-pain conditions (*p* = 0.256).

##### Intention to Help

There was a significant effect of condition on participants’ responses, *F* (1.292, 40.041) = 44.85, *p* < 0.001, *η*^2^ = 0.673. Pairwise comparisons showed statistically significant differences between non-painful and the other two conditions (*p*s < 0.001). Participants reported to have almost similar empathic behavior toward a stranger suffering from either physical or psychological painful incidents. On the other hand, on the non-painful condition, they indicated they will “*probably help*” or “*don’t know*” if they will help.

##### Interaction Between Loss Experience and Conditions

The final analysis focused on the interaction between participants’ past experience of psychological pain and conditions. Accordingly, there was a significant multivariate interaction effect, Wilks’ *λ* = 0.291, *F* (10, 22) = 5.35, *p* = 0.001, *η*^2^ = 0.71, as the follow-up univariate analysis indicated the only significant difference was observed in the reported perspective taking ability, *F* (2, 62) = 6.290, *p* = 0.003, *η*^2^ = 0.169. Participants in the *Loss* group reported to have higher perspective taking for the individuals in the psychological-pain condition following by the non-painful condition. On the contrary, the *No-Loss* group showed a reverse pattern meaning they could take the perspective of protagonists in the non-painful condition more than psychological-pain condition. Both groups reported to have almost similar perspective taking for the individuals suffering from one sort of physical pain. On average, participants in the *Loss* group reported to have higher perspective taking in all conditions especially in the psychological-pain condition.

**Figure 3:**
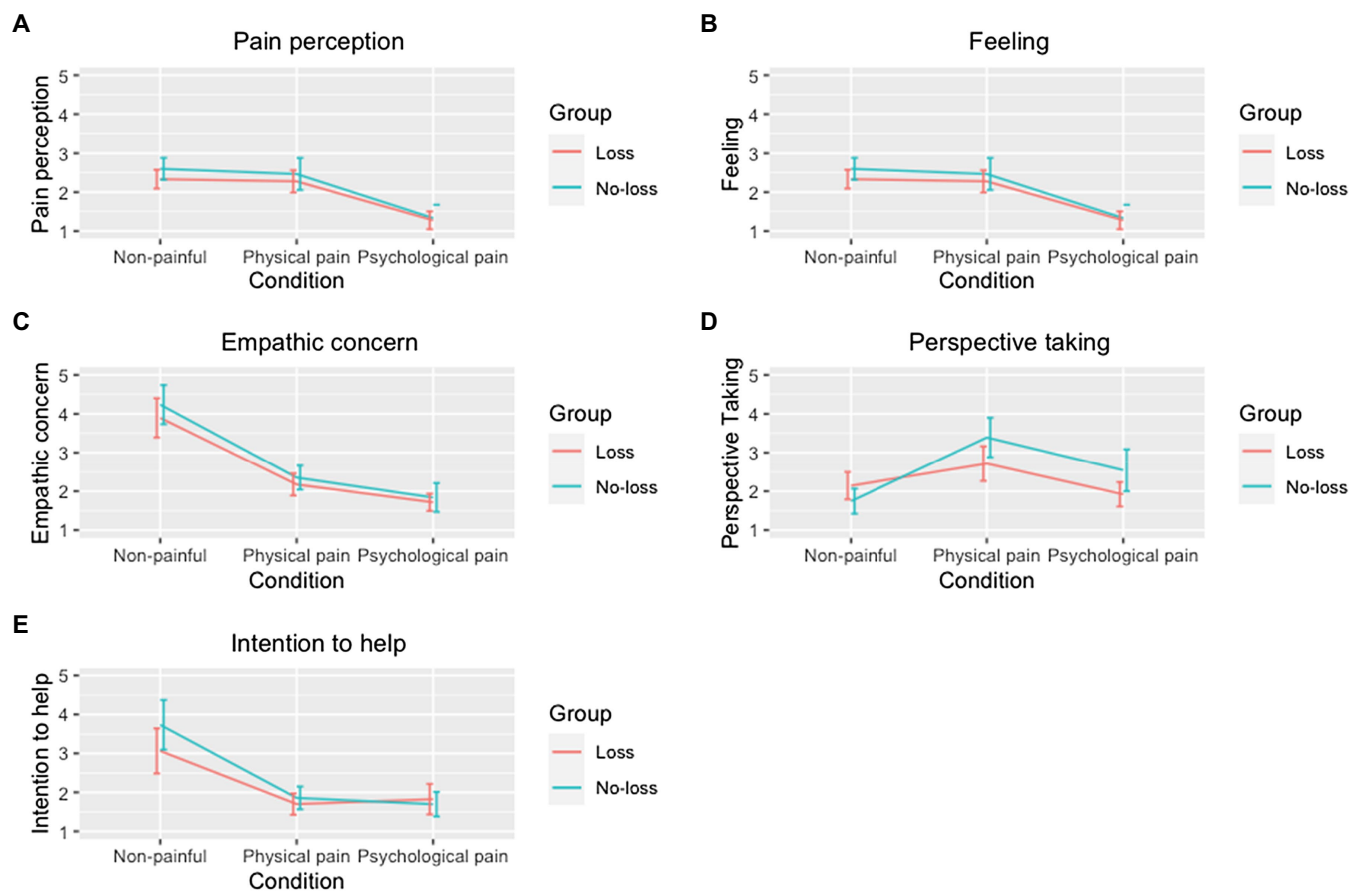
Group comparison based on conditions. **(A)** Pain perception. **(B)** Intensity of feeling. **(C)** Empathic concern. **(D)** Perspective taking. **(E)** Intention to help. Each line plot compares the responses per condition and group within each variable. In the Y-axis, 1 means the strongest response (e.g., the greatest pain judgment), while 5 means the weakest response (e.g., the weakest pain judgment).

For the other dependent variables, the interaction between group affiliation and condition did not reach statistical significance: pain perception, *F* (2, 62) = 1.025, *p* = 0.365, *η*^2^ = 0.032; feeling, *F* (1.654, 51.263) = 0.465, *p* = 0.594, *η*^2^ = 0.015; empathic concern, *F* (1.107, 34.312) = 0.228, *p* = 0.661, *η*^2^ = 0.007, and intention to help, *F* (1.292, 40.041) = 2.953, *p* = 0.084, *η*^2^ = 0.087. See Figure 3 for details.

### ERP Analysis

#### Frontal Region ERP Analysis

We performed the mixed-effects analysis and electrode-wise comparison within seven electrodes in frontal regions. First, when we analyzed the ELPP, all main effects of condition, *F* (2, 2421.91) = 0.07, *p* = 0.93, group, *F* (1, 38.07) = 0.45, *p* = 0.51, electrode location, *F* (4, 2382.66) = 0.97, *p* = 0.42, and all interaction effects of condition × group, *F* (2, 2422.50) = 0.40, *p* = 0.67, electrode location × condition, *F* (8, 2382.66) = 0.16, *p* = 1.00, electrode location × group, *F* (4, 2382.66) = 1.72, *p* = 0.14, and electrode location × group × condition, *F* (8, 2382.66) = 0.34, *p* = 0.95, were non-significant.

Second, in the analysis of the LLPP, we found only the significant main effect of electrode location, *F* (4, 2356.17) = 6.36, *p* < 0.001. All other main effects of condition, *F* (2, 2372.43) = 1.31, *p* = 0.27, and group, *F* (1, 26.57) = 0.04, *p* = 0.84, and interaction effects of condition × group, *F* (2, 2378.82) = 1.91, *p* = 0.15, electrode location × condition, *F* (8, 2356.17) = 0.28, *p* = 0.97, electrode location × group, *F* (18, 2356.17) = 0.48, *p* = 0.75, and electrode location × group × condition, *F* (8, 2356.17) = 0.45, *p* = 0.89, were non-significant.

Third, for the analysis of the VLLPP, the main effect of electrode location, *F* (4, 2352.95) = 4.04, *p* < 0.01, and the interaction effect of group × condition, *F* (2, 2388.43) = 4.04, *p* < 0.05, were significant. However, the main effects of condition, *F* (2, 2380.99) = 1.62, *p* = 0.20, group, *F* (1, 14.78) = 0.29, *p* = 0.60, and the interaction effects of electrode location × condition, *F* (8, 2352.95) = 0.86, *p* = 0.55, electrode location × group, *F* (4, 2352.05) = 0.35, *p* = 0.85, and electrode location × group × condition, *F* (8, 2352.95) = 0.64, *p* = 0.74, were non-significant.

#### Whole-Brain ERP Analysis

In addition, we explored whole-brain ERP analysis for each time window. First, we conducted the whole-brain ERP analysis within the ELPP. In this analysis, the main effects of condition, *F* (2, 9314.30) = 8.23, *p* < 0.001, and electrode location, *F* (18, 9591.00) = 133.99, *p* < 0.001, were significant. The main effect of group, *F* (1, 44.90) = 3.56, *p* = 0.07, and all interaction effects, including condition × group, *F* (2, 9331.80) = 0.62, *p* = 0.96, electrode location × condition, *F* (36, 9591.00) = 0.62, *p* = 0.96, electrode location × group, *F* (18, 9591.00) = 1.52, *p* = 0.07, and electrode location × group × condition, *F* (36, 9591.00) = 0.34, *p* = 1.00, were non-significant.

Second, from the analysis of the LLPP, we found the significant main effects of condition, *F* (2, 9394.60) = 12.10, *p* < 0.001, and electrode location, *F* (18, 9593.30) = 33.44, *p* < 0.001, and interaction effect of condition × group, *F* (2, 9416.0) = 3.34, *p* < 0.05. All other main effects of group, *F* (1, 31.00) = 2.47, *p* = 0.20, and interaction effects of electrode location × condition, *F* (36, 9593.30) = 0.962, *p* = 0.96, electrode location × group, *F* (18, 9593.30) = 0.97, *p* = 0.49, and electrode location × group × condition, *F* (36, 9593.30) = 0.37, *p* = 1.00, were non-significant.

Third, when we examine the VLLPP, the main effects of condition, *F* (2, 9638.00) = 4.69, *p* < 0.01, electrode location, *F* (18, 9582.00) = 9.42, *p* < 0.001, and the interaction effect of group × condition, *F* (2, 9642.50) = 9.20, *p* < 0.001, were significant. However, the main effects of group, *F* (1, 18.50) = 1.38, *p* = 0.26, and the interaction effects of electrode location × condition, *F* (36, 9582.00) = 0.68, *p* = 0.93, electrode location × group, *F* (18, 9582.00) = 0.50, *p* = 0.96, and electrode location × group × condition, *F* (36, 9582.00) = 0.83, *p* = 0.75, were non-significant.

#### ERP Analysis Within Each Electrode

We analyzed ERP within each electrode with mixed-effects analysis. The analysis was performed within (1) seven hypothesize frontal electrodes and (2) the whole brain. In the cases of the ELPP and LLPP, we could not find any electrode location that showed at least one significant main or interaction effect from both the analysis within seven frontal electrodes and the whole-brain analysis when the false discovery rate correction was applied.

**Figure 4:**
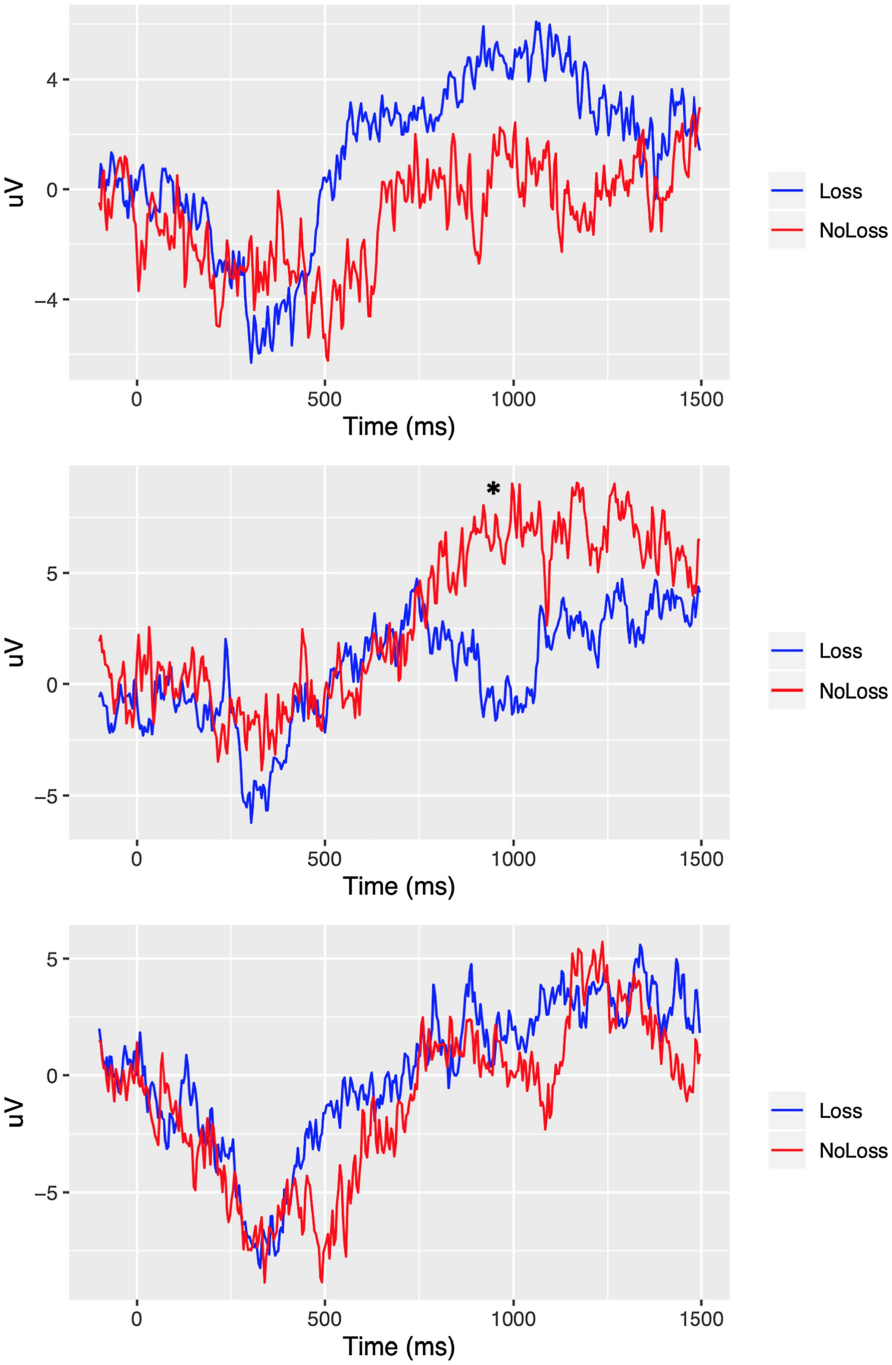
ERP comparison in Fp2 between *Loss* versus *No-Loss* groups. Top: physical-pain condition. Middle: psychological-pain condition. Bottom: non-painful condition. ^*^ represents a significant different at *p* < 0.05 (FDR adjusted).

**Figure 5:**
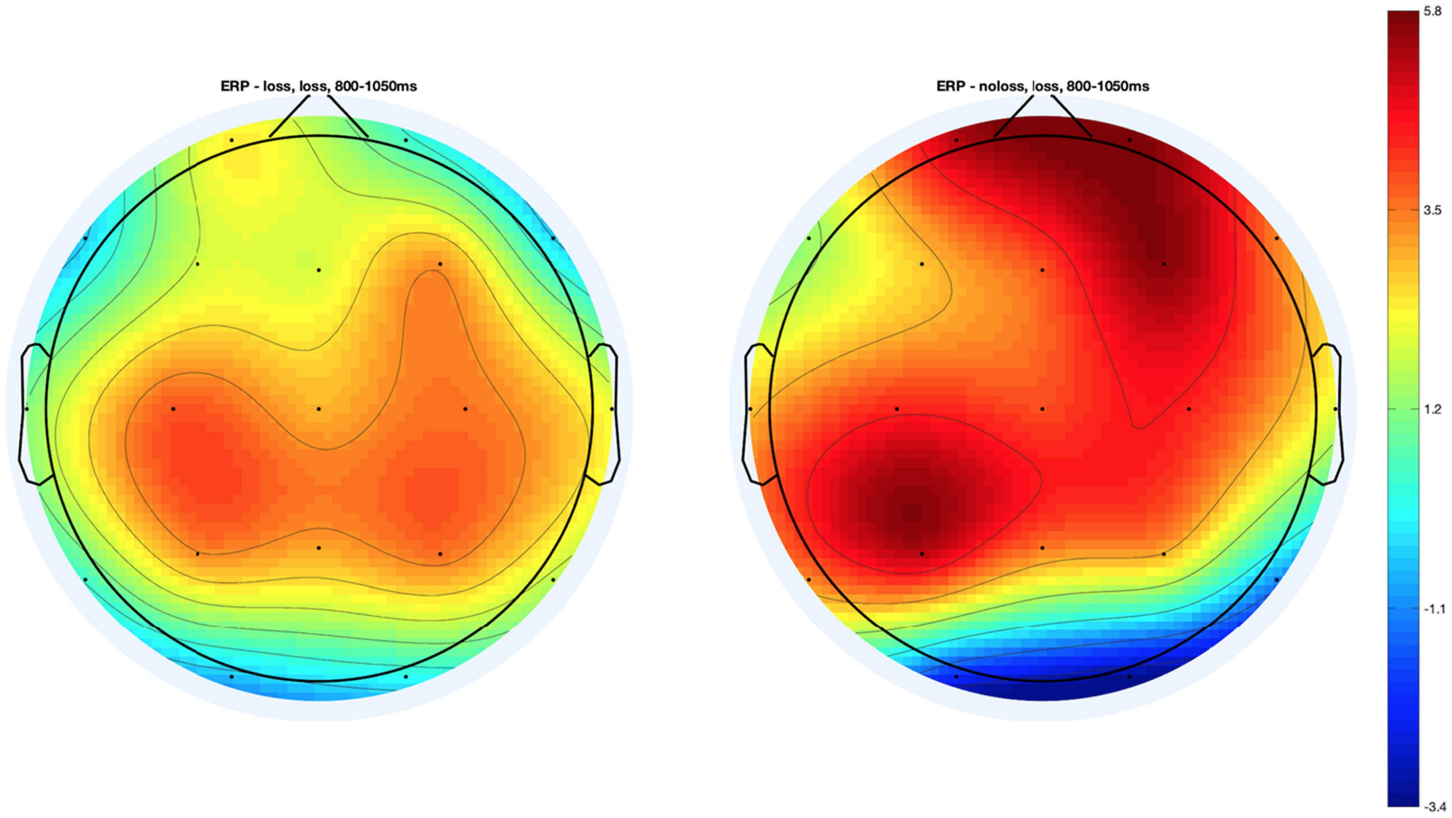
Topography maps of the very late Late Positive Potential (VLLPP) in the *Psychological-pain* condition in two groups.

In the case of the VLLPP, we found the significant interaction effect of group × condition only in Fp2 from both the analysis of frontal electrodes, *F* (2, 425.09) = 5.01, *p* < 0.01, *q* = 0.02, and the whole-brain analysis, *p* < 0.01, *q* = 0.05 (see Figure 4). When we performed the additional *t*-tests, we found that participants in the *Loss* group showed significantly lower ERP in Fp2 compared with those in the *No-Loss* group in the psychological-pain condition, *t*(137.45) = −2.40, *p* < 0.05, *D* = 0.38. However, such ERP difference was non-significant in the physical-pain condition, *t*(151.43) = 1.64, *p* = 0.10, *D* = 0.25, and non-painful condition, *t*(153.94) = 0.34, *p* = 0.73, *D* = 0.05 (see Figure 4). The scalp topography of the VLLPP in the *Loss* condition in both groups is presented in Figure 5 for readers’ information. For additional information, the mean ERPs within the defined time windows across groups are summarized in Supplementary Tables S1 (for the physical-pain condition), S2 (for the psychological-pain condition), and S3 (for the non-painful condition).

### Exploratory Correlational Analysis

Given that we found significant ANOVA and electrode-wide analysis results only from the analysis of VLLPP, we focused on this time window in exploratory correlational analysis. The result showed a significant association (2log*BF* ≥ 2) between ERPs, behavioral responses, and IRI subscale scores in the physical-pain condition. Significant correlations were found in these pairs: F4 and pain perception, *r* = 0.40, 2log*BF* = 2.64; T3 and intention to help, *r* = 0.39, 2log*BF* = 2.29; C4 and IRI-PT, *r* = −0.39, 2log*BF* = 2.31; and Cz and IRI-PD, *r* = 0.40, 2log*BF* = 2.60.

## Discussion

This study compared the empathic behavior (in forms of dispositional and situational empathy) and neural correlation of participants toward observing strangers in painful (physical and psychological) and non-painful conditions. In addition, the effect of having prior experience of psychological pain on empathic reactions was assessed. For this study, participants were grouped into *Loss* and *No-Loss* based on prior experience of grief. As the result of behavioral analysis indicated, prior experience of psychological pain did not affect participants’ dispositional empathy as observed in self-reported questionnaire. Both groups reported to have higher affective empathy than cognitive empathy. This result was in line with previous study in which American college students answered the IRI self-reported questionnaire and had a higher score on affective empathy (Yaghoubi Jami et al., 2019). On the other hand, having first-hand experience of psychological pain did affect situational cognitive empathy; those with an experience of psychological pain reported to have higher ability in understanding another individual in psychologically painful condition. Moreover, ERP analysis suggested that participants with prior loss experience demonstrated significantly lower activity in right frontal region, Fp2, within VLLPP time window in the psychological-pain condition compared with those without prior loss experience.

Participants’ empathic responses when observing different types of pictures support the validity of this study. As expected, non-painful pictures received the lowest ratings in all aspects of empathic reaction. The pictures in this category were intended to trigger no pain, no negative emotion, and no empathic concern, and there was no need for participants to help the pictures’ protagonists, as there was no suffering involved in the pictures of this category. Given the nature of pictures and participants’ reactions in this category, it could be concluded that this study was successful in capturing people’s reaction to different types of pain.

### Discussion on Behavioral Analysis Results

#### Conditions and Aspects of Situational Empathy

As expected, the *loss*/*no-loss* condition affected participants’ empathic responsiveness across assessments. Regardless of prior experience of psychological pain, all participants reported feeling higher levels of pain after observing another grieving individual than witnessing others’ physical pain. The findings that feeling more pain from others’ psychological pain along with feeling almost no pain after observing a non-painful picture suggest that participants could accurately judge the situation and had a precise understanding of the other person’s physical and emotional states. Similar results were found in previous studies in which participants with and without prior experience of pain reported similar amount of pain resulted from observing others’ pain (e.g., Batson et al., 1981; Yaghoubi Jami et al., 2021). Unlike the position advanced by Nordgren et al. (2011), our findings suggest that having a first-hand painful experience is not necessarily linked to an accurate estimation of others’ emotional pain. However, it is possible that having a similar pain perception may not lead to empathic responsiveness; therefore, other aspects of empathy (e.g., feeling, empathic concern, and perspective taking) need to be considered.

The reported feelings after observing different types of pictures were interesting; sadness, distressed, and happy were the most frequent feelings reported after observing psychological, physical, and non-painful pictures, respectively. This result is consistent with previous studies in which similar emotions were evoked by different painful stimuli (Yaghoubi Jami et al., 2021). Feeling sadness for others’ grief is not a surprising emotion as psychological pain is recalled as the “most negative experience in life” (Jaremka et al., 2011, p. 46). Similarly, psychological-pain condition triggered the highest levels of empathic concern as well as intention to help compared to the other two conditions. Considering the non-significant effect of prior experience of psychological pain on participants’ dispositional empathy (i.e., self-reported questionnaire), it is not surprising to observe both groups rated the same amount of empathic concern and were willing to alleviate the suffering of strangers in psychological pain.

#### Prior Psychological Pain Experience and Situational Empathy

The only aspect of empathy affected by prior experience of psychological pain was participants’ ability in taking others’ perspective. That is, those who had lost a loved one were more able to understand the pain caused by similar incidents. Recently, Yaghoubi Jami et al. (2021) argued that a comprehensive understanding of psychological pain is attributed to the observer’s personal experiences regarding similar sources of pain. Accordingly, an observer’s empathic response to someone else’s pain is tied to their personal experience of the same type of pain. This finding suggests that although pain can come in a variety of forms – pain as a result of a physical injury, social isolation, or losing a loved one (Eisenberger, 2012) – it may be that experience of pain in one context enhances the individual’s ability to experience empathy, but not in another painful context. For example, an individual with experience of social exclusion could feel the pain of another individual in a similar situation (i.e., social isolation) but might have limited understanding of grieving person’s pain and suffering. Given the rationale proposed by Hoffman (2001) for how having shared experience could facilitate empathy by bringing back memories and feeling associated with such experiences, it seems that such experiences helped participants to develop a more accurate understanding of similar traumas and be more aware of emotional states caused by this type of pain.

### Discussion on ERP Analysis Results

#### Whole-Brain ERP Analysis

In our ERP analysis, as expected we found significant difference in the VLLPP in Fp2 between the *Loss* versus *No-Loss* groups in the psychological-pain condition. This result suggests that the prior experience of grief may be associated with the brain activity in the frontal region near Fp2. According to Hajcak et al. (2010), the increased VLLPP is associated with the increased intensity of emotional arousal after watching pleasant or unpleasant visual stimuli. In addition, prior research has shown that right frontal region, which is connected with Fp2, is related to emotional aspects of empathic responses to painful situations (Decety and Jackson, 2004; Leigh et al., 2013; Reniers et al., 2014). Furthermore, more directly, Maratos et al. (2000) reported that the LPPs in an electrode attached on the right prefrontal region, which corresponds to Fp2 in the present study, were significantly associated with perception of novel negative emotional stimuli; the LPPs in the same electrode were significantly smaller while perceiving old stimuli. The findings from Maratos et al. (2000) might suggest that the right prefrontal region corresponding to Fp2, which was analyzed in the present study, shall be focused while analyzing the LPPs associated with emotional perception. Given these, the relatively decreased Fp2 VLLPP among participants who experienced prior psychological pain perhaps suggests that such participants might be less strongly aroused by visual stimuli presenting others’ loss compared with participants who did not experience any prior loss.

One explanation for less emotional arousal for people with an experience of psychological pain could be linked to emotional numbness. Experiencing a negative emotional state, such as grief, could affect people capacity in being sensitive toward others’ pain as well as their pain tolerance (Twenge et al., 2007). This alteration may serve as a survival mechanism for the grieving individual; individuals protect themselves from further harm by becoming emotionally numb or detached. Therefore, these individuals might not be as aroused as those without such painful experience when they see another individual in the same situation. Nevertheless, less emotional arousal does not mean they cannot be empathic, or their empathic behavior is decreased as a result of their experience. This argument can be supported by the result of our behavioral analysis and a previous study in which participants stated that their painful experience actually helped them acquire a deeper understanding toward the person in the same situation (Yaghoubi Jami et al., 2021).

Interestingly, the observed decrease in the VLLPP in Fp2, which is associated with emotional empathic responses to pain (Decety and Jackson, 2004; Leigh et al., 2013; Reniers et al., 2014), among participants in the *Loss* group is consistent with the findings from behavioral analysis and prior research on loss. At the behavioral level, the *Loss* group showed significantly higher perspective taking in the psychological-pain condition as compared to the *No-Loss* group. However, there was no difference in affective empathy or emotional arousal in the psychological-pain condition between the two groups.

Previous behavioral studies have indicated that although people are often significantly depressed after bereavement, a negative emotional state is likely to be negated in 1–2 months (for review, see Dutton and Zisook, 2005). Moreover, Preis and Kroener-Herwig (2012) reported that having previous painful experience was significantly positively associated with perspective taking; however, the same experience was not significantly associated with emotional reaction. Given this previous research, it may be that people experience emotional and cognitive adaptation after loss. Therefore, in the current study, participants who experienced loss did not necessarily show increased affective arousal, but showed more cognitively sophisticated reaction, such as perspective taking, in the psychological-pain condition. It is interesting to note that our findings at the neural level are consistent with this view since the eventual adaptation to loss would be associated with decreased VLLPP activity in Fp2.

#### Correlation Between ERP and Behavioral Responses

Interestingly, the VLLPP analyses did not indicate any significant correlations in the psychological-pain condition. Given that the ERP in Fp2 in this condition was significantly different between *Loss* and *No-Loss* groups, it may be that the ERP in processing this type of stimuli, the psychological pain, might be more closely associated with prior experience rather than behavioral responses or dispositional empathy. This relationship was reversed in the physical-pain condition, which was supposed to present physical-pain stimuli that might be perceived to be vivid to all participants regardless of their prior experience of pain.

As argued by Nordgren et al. (2011), people may have a better understanding of others’ psychological pain only if they experienced the same pain. This effect is likely the result of remembering one’s own experiences accompanied by associated emotions (Hoffman, 2001). However, when encountering a person experiencing the agony of grief, most people would be motivated to help in preventing the life-threating consequences of complicated grief (Bosticco and Thompson, 2005; Mee et al., 2006; Goodrum, 2008). As de Waal (2010, p. 124) stated, “advanced empathy requires both mental mirroring and mental separation”; therefore, to efficiently help someone who needs us, one needs to be aware of self-other boundaries and mentally separate their emotional state from the other individual to behave empathically.

### Limitations and Suggestions for Future Research

Each study has some limitations that could threaten the generalizability of its results and interpretations. The current study is no exception, and our results should be interpreted with caution especially before making any generalizations to the larger population. Perhaps, the most important limitation of the present study is related to sample size in each group. As stated previously and given the exploratory nature of the study, we determined the sample size based on similar published studies. However, we noted that the number of relevant studies was not large, which limited the guidance they provided. Our results may help highlighting the needs for conducting more studies on empathic behavior and psychological pain to better estimate the required sample size.

In addition to sample size concerns, we would note that there were unequal numbers of participants in each group. Although the sample size inequality was very small and any characteristic differences between each group were controlled for in the analysis, still it may be that the unbalanced groups affected the observed results. Therefore, replicating this study with the goal of increasing the power of analysis could be a promising area for future studies.

Another limitation was related to unbalanced number of female and male participants in each group. As noted previously, there were more female participants in each group. Although the present study applied Type III Sum Squares, the suggested analytical approach for unbalanced sample sizes (Pituch and Stevens, 2015), the present study failed to explore gender differences in participants’ responses. There is numerous theoretical and empirical evidence showing gender effect on empathic responses (Yaghoubi Jami et al., 2019); thus, future studies may benefit from having balanced number of male and female participants in exploring the relationship between prior psychological pain and empathy.

Stimulus selection could also pose a limitation to this study. Specifically, the number of trials in each condition was limited, which could affect the result and interpretation. Considering the existing database for psychological pain has been used in only one previous study (Yaghoubi Jami et al., 2021), it was not possible to include more validated pictures in each condition. Future studies can follow the same procedure of validation with a different picture pool and create a larger standardized picture database.

Finally, the EEG recording system has some limitations. This study used a 21-channel EEG; therefore, the recorded data are limited. Although there is no clear guideline regarding the minimum required number of channels for ERP analysis, some have suggested that robust ERP sources are efficiently captured with an EEG system using 35 channels (Lau et al., 2012). Thus, further investigations employing an EEG system with more electrodes might be beneficial to address any signal quality-related issue that might exist in the current study.

## Conclusion

In the present study, we examined participants’ behavioral responses to different types of painful (in the form of physical and psychological pain), and non-painful contexts and the neural correlates associated with these contexts. Our statistical analyses focused on the associations between the stimulation type, prior painful experience, behavioral responses, and ERPs. The findings from both the behavioral and neural components of the study demonstrated that empathic reactions—in form of pain intensity, subjective feeling, empathic concern, perspective taking, and intention to help—were dependent on the type of conditions. In addition, the presence of prior psychological painful experience was also significantly associated with the differences in behavioral and neural responses particularly in cognitive empathy. These findings provide useful insights about directions for future research examining factors associated with empathic responses in painful situations.

## Data Availability Statement

The raw data supporting the conclusions of this article will be made available by the authors, without undue reservation.

## Ethics Statement

The studies involving human participants were reviewed and approved by the Institutional Review Board of the University of Alabama. The patients/participants provided their written informed consent to participate in this study.

## Author Contributions

PJ, ST, BM, and RH contributed to conception and design of the study. PJ conducted the experiments and collected the data. PJ and HH performed the statistical analysis and wrote the first draft of the manuscript. All authors contributed to manuscript revision, read, and approved the submitted version.

### Conflict of Interest

The authors declare that the research was conducted in the absence of any commercial or financial relationships that could be construed as a potential conflict of interest.

## Footnotes

^1^https://www.neurobs.com

^2^http://sccn.ucsd.edu/eeglab

^3^https://bio-medical.com/wineeg-advanced-software-for-mitsar
