# Supplementary material for: Do Histories of Painful Life Experiences Affect the Expression of Empathy Among Young Adults? An Electroencephalography Study
Source: Front Psychol. 2021 Jul 16;12:689304. doi: 10.3389/fpsyg.2021.689304 (PMC8322231; doi:10.3389/fpsyg.2021.689304)
Supplement: Supplementary file 1 [file Data_Sheet_1.PDF]

Table S1

Mean ERPs in designated times windows in the physical pain condition

Time Window	Electrode	Loss group		No-loss group		All	
		Mean	SD	Mean	SD	Mean	SD
ELPP	Fp1	-2.70	12.59	1.53	17.34	-.82	14.99
	Fp2	-3.10	12.67	-3.31	15.43	-3.19	13.93
	F7	-3.76	14.29	-2.81	12.43	-3.34	13.47
	F3	-4.17	12.50	-5.07	13.24	-4.57	12.80
	Fz	-4.44	12.80	-4.37	13.18	-4.41	12.93
	F4	-4.61	12.16	-4.57	13.32	-4.60	12.65
	F8	-5.13	11.72	-4.25	13.81	-4.74	12.66
	T3	-2.82	11.18	-1.70	10.33	-2.32	10.80
	C3	-2.00	12.45	-2.89	11.68	-2.40	12.09
	Cz	-2.93	13.44	-4.11	13.30	-3.45	13.35
	C4	-2.24	11.65	-2.56	12.26	-2.38	11.89
	T4	-1.51	9.94	-2.48	9.98	-1.95	9.94
	T5	3.76	12.64	2.46	10.82	3.18	11.85
	P3	2.37	14.13	2.58	12.30	2.47	13.31
	Pz	-.15	14.44	.44	13.00	.11	13.79
	P4	3.19	13.19	3.12	11.61	3.16	12.48
	T6	3.94	11.04	7.70	13.99	5.61	12.54
	O1	7.34	13.77	7.54	13.36	7.43	13.55
	O2	8.15	13.93	8.64	13.03	8.37	13.50
LLPP	Fp1	1.47	14.00	1.99	18.47	1.70	16.10
	Fp2	2.52	14.67	-1.06	17.54	.93	16.06
	F7	-2.33	14.84	-3.91	17.07	-3.04	15.84
	F3	1.82	13.92	-2.54	15.47	-.12	14.75
	Fz	1.89	13.46	-.23	16.18	.95	14.73
	F4	2.93	13.43	-1.30	14.95	1.05	14.24
	F8	-.09	12.63	-2.56	17.35	-1.19	14.93
	T3	.59	13.57	.33	11.43	.47	12.63
	C3	3.82	14.22	1.74	15.03	2.89	14.59
	Cz	3.44	14.37	1.87	16.69	2.74	15.42
	C4	5.91	12.76	2.63	14.62	4.45	13.68
	T4	3.78	11.07	.11	12.03	2.14	11.62
	T5	6.11	11.99	4.12	12.98	5.22	12.45
	P3	7.73	15.05	7.63	15.94	7.68	15.41
	Pz	7.35	15.62	7.74	17.89	7.53	16.62
	P4	9.53	14.18	7.90	15.13	8.80	14.59
	T6	5.25	11.75	5.56	14.45	5.39	12.98
	O1	4.79	13.61	4.27	14.61	4.56	14.03
	O2	6.32	13.78	5.27	14.10	5.86	13.89
VLLPP	Fp1	2.94	14.04	3.64	19.95	3.25	16.88

Fp2	4.35	14.45	.31	18.04	2.55	16.22
F7	1.05	14.98	.89	18.27	.98	16.48
F3	5.65	12.84	2.64	15.50	4.31	14.12
Fz	5.04	13.42	5.74	16.23	5.35	14.70
F4	5.34	13.39	4.59	14.82	5.01	14.01
F8	3.78	13.27	.46	17.66	2.30	15.43
T3	3.41	12.77	2.98	11.96	3.22	12.39
C3	5.02	13.01	5.57	15.14	5.26	13.96
Cz	4.32	12.48	5.82	16.36	4.99	14.32
C4	5.76	11.27	5.88	14.37	5.82	12.71
T4	3.83	11.25	1.94	10.84	2.99	11.08
T5	2.36	11.23	.57	12.51	1.56	11.82
P3	4.44	12.54	4.55	16.13	4.49	14.21
Pz	3.59	13.09	5.32	18.04	4.36	15.47
P4	4.20	12.84	4.59	15.88	4.37	14.24
T6	-.48	11.88	.13	13.53	-.21	12.61
O1	-1.85	11.58	-2.40	15.38	-2.10	13.37
O2	-1.12	12.01	-1.43	14.58	-1.26	13.18

Table S2

Mean ERPs in designated times windows in the psychological pain condition

Time Window	Electrode	Loss group		No-loss group		All	
		Mean	SD	Mean	SD	Mean	SD
ELPP	Fp1	-1.30	14.64	-1.49	18.52	-1.39	16.50
	Fp2	-2.00	13.36	-1.85	15.77	-1.93	14.48
	F7	-1.86	13.03	-1.34	14.34	-1.62	13.62
	F3	-3.45	12.35	-4.27	11.81	-3.83	12.07
	Fz	-3.24	12.10	-5.85	10.88	-4.45	11.59
	F4	-4.05	10.75	-4.18	12.13	-4.11	11.38
	F8	-4.89	12.51	-4.44	13.49	-4.69	12.93
	T3	-2.65	12.05	-2.07	12.38	-2.38	12.17
	C3	-1.92	11.34	-4.19	13.43	-2.97	12.36
	Cz	-2.12	12.33	-4.68	13.93	-3.30	13.11
	C4	-3.26	11.74	-3.00	12.72	-3.14	12.17
	T4	-2.95	10.78	-2.63	12.24	-2.80	11.45
	T5	2.81	11.03	5.15	15.21	3.90	13.15
	P3	2.66	12.55	2.33	14.54	2.50	13.47
	Pz	1.19	13.42	-.26	15.87	.52	14.58
	P4	3.01	12.78	3.63	14.36	3.30	13.50
	T6	5.46	14.04	7.82	14.56	6.56	14.28
	O1	7.63	13.20	7.35	16.51	7.50	14.78
	O2	8.48	13.21	9.07	14.05	8.75	13.57
LLPP	Fp1	1.12	15.70	.39	19.40	.78	17.46
	Fp2	2.17	12.44	1.78	17.52	1.99	14.96
	F7	-2.45	13.06	-5.01	18.81	-3.64	15.98
	F3	-.95	11.40	-2.59	15.60	-1.71	13.49
	Fz	.53	12.25	-2.17	15.41	-.72	13.82
	F4	.98	12.11	-.13	15.52	.47	13.77
	F8	-.53	15.06	-2.54	15.66	-1.46	15.32
	T3	-.98	13.16	-.92	11.10	-.95	12.21
	C3	2.72	12.37	.64	13.43	1.76	12.87
	Cz	2.56	13.98	.57	16.00	1.64	14.94
	C4	3.76	14.37	1.09	14.36	2.52	14.38
	T4	2.79	12.41	-.19	12.57	1.41	12.53
	T5	5.49	13.34	6.99	13.06	6.18	13.19
	P3	6.67	15.49	8.14	14.52	7.35	15.02
	Pz	6.53	17.05	6.97	15.95	6.73	16.50
	P4	8.36	16.07	7.38	14.90	7.91	15.50
	T6	7.88	15.04	5.66	13.24	6.85	14.24
	O1	5.56	15.51	5.39	16.69	5.48	16.02
	O2	6.81	15.19	5.02	13.81	5.98	14.55
VLLPP	Fp1	1.31	19.37	5.34	19.46	3.18	19.46
	Fp2	.33	13.65	6.44	18.23	3.16	16.18
	F7	-.14	15.01	.63	18.40	.22	16.62
	F3	1.51	12.12	3.41	15.96	2.39	14.02

Fz	1.40	13.29	4.34	16.89	2.76	15.09
F4	2.37	13.78	6.02	16.14	4.06	14.99
F8	.52	15.01	3.45	16.71	1.88	15.84
T3	1.28	13.45	3.09	11.95	2.12	12.77
C3	3.46	12.39	4.77	13.96	4.06	13.12
Cz	2.83	13.96	4.59	17.17	3.65	15.51
C4	3.53	13.90	4.96	14.80	4.19	14.30
T4	2.50	13.03	2.56	13.22	2.52	13.08
T5	.84	13.62	4.34	14.47	2.46	14.09
P3	2.91	14.81	6.26	15.12	4.46	15.01
Pz	3.08	16.52	4.61	16.97	3.79	16.69
P4	3.93	15.33	4.10	17.67	4.01	16.40
T6	2.51	13.84	-.06	15.63	1.32	14.70
O1	-.41	14.46	-.96	17.09	-.66	15.69
O2	.38	14.56	-1.78	15.76	-.62	15.12

Table S3

Mean ERPs in designated times windows in the non-painful condition

Time Window	Electrode	Loss group		No-loss group		All	
		Mean	SD	Mean	SD	Mean	SD
ELPP	Fp1	-2.62	14.14	-3.65	17.02	-3.10	15.51
	Fp2	-4.28	13.99	-5.08	12.39	-4.65	13.23
	F7	-3.61	13.24	-3.66	14.80	-3.64	13.95
	F3	-3.75	11.40	-5.05	13.48	-4.36	12.39
	Fz	-3.21	12.15	-5.84	12.83	-4.44	12.51
	F4	-4.10	11.93	-5.19	13.80	-4.61	12.81
	F8	-4.64	13.73	-4.66	11.68	-4.65	12.77
	T3	-2.35	10.40	-3.22	12.05	-2.76	11.18
	C3	-.63	10.39	-3.84	13.26	-2.13	11.89
	Cz	-1.24	11.53	-4.43	13.38	-2.73	12.49
	C4	-2.12	11.07	-4.23	10.94	-3.10	11.03
	T4	-3.37	10.90	-3.81	10.12	-3.57	10.52
	T5	3.85	10.60	2.14	11.75	3.05	11.15
	P3	3.29	11.32	.47	12.54	1.97	11.95
	Pz	-.02	13.58	-1.72	12.32	-.82	12.99
	P4	3.25	11.59	.96	10.75	2.18	11.23
	T6	4.06	10.48	4.69	13.57	4.36	11.99
	O1	6.82	11.33	6.07	11.19	6.47	11.24
	O2	7.30	13.08	5.92	12.58	6.65	12.83
LLPP	Fp1	.43	16.95	-1.45	15.55	-.45	16.29
	Fp2	.04	14.68	-1.87	14.68	-.85	14.67
	F7	-4.36	15.88	-1.79	15.87	-3.16	15.88
	F3	-1.36	13.45	-1.82	13.30	-1.57	13.34
	Fz	-.27	13.02	-.99	12.82	-.61	12.89
	F4	1.27	14.40	-1.18	13.22	.12	13.88
	F8	-.33	15.71	-.73	14.22	-.52	14.99
	T3	-1.77	14.58	-.92	12.07	-1.37	13.43
	C3	.94	13.67	.83	12.15	.89	12.94
	Cz	1.35	13.69	1.82	13.76	1.57	13.68
	C4	2.80	13.80	1.66	12.20	2.27	13.05
	T4	-.13	13.37	.16	9.71	.00	11.77
	T5	3.87	12.73	4.33	9.55	4.08	11.32
	P3	4.85	14.14	5.35	11.58	5.08	12.97
	Pz	3.05	15.88	5.05	13.88	3.98	14.97
	P4	6.43	14.82	6.30	12.61	6.37	13.79
	T6	4.35	13.00	5.34	10.33	4.81	11.80
	O1	3.21	12.69	5.03	10.05	4.06	11.53
	O2	3.62	13.05	3.88	11.85	3.74	12.47
VLLPP	Fp1	3.90	16.57	4.43	20.20	4.15	18.30
	Fp2	1.75	15.06	.88	17.74	1.34	16.32
	F7	.95	17.54	3.94	18.46	2.34	17.98
	F3	2.22	12.67	2.16	14.59	2.19	13.56

Fz	2.57	12.64	2.71	13.57	2.64	13.04
F4	3.87	14.29	2.92	14.03	3.43	14.13
F8	1.81	14.95	3.03	15.13	2.38	15.00
T3	1.44	14.48	1.92	13.50	1.66	13.99
C3	3.35	13.08	3.40	12.36	3.37	12.71
Cz	3.84	14.33	4.19	14.07	4.00	14.17
C4	4.14	14.20	3.23	13.27	3.71	13.74
T4	1.20	14.51	1.25	11.88	1.22	13.31
T5	2.38	12.71	.72	11.27	1.61	12.05
P3	3.51	14.20	2.48	12.99	3.03	13.62
Pz	2.47	15.82	2.49	14.90	2.48	15.35
P4	4.03	14.99	2.39	14.37	3.26	14.69
T6	.69	13.11	-.12	13.18	.31	13.11
O1	-.43	12.02	-.55	11.61	-.49	11.79
O2	-1.01	13.12	-1.80	12.51	-1.38	12.81
